# Combined renin-angiotensin-aldosterone system blockade and statin therapy effectively reduces the risk of cerebrovascular accident in autosomal dominant polycystic kidney disease: a nationwide population-based cohort study

**DOI:** 10.18632/oncotarget.18636

**Published:** 2017-06-27

**Authors:** Pei-Hsun Sung, Hsin-Ju Chiang, Mel S. Lee, John Y. Chiang, Hon-Kan Yip, Yao-Hsu Yang

**Affiliations:** ^1^ Division of Cardiology, Department of Internal Medicine, Kaohsiung Chang Gung Memorial Hospital and Chang Gung University College of Medicine, Kaohsiung, Taiwan; ^2^ Department of Obstetrics and Gynecology, Kaohsiung Chang Gung Memorial Hospital and Chang Gung University College of Medicine, Kaohsiung, Taiwan; ^3^ Chung Shan Medical University School of Medicine, Taichung, Taiwan; ^4^ Department of Orthopedics, Kaohsiung Chang Gung Memorial Hospital and Chang Gung University College of Medicine, Kaohsiung, Taiwan; ^5^ Department of Computer Science and Engineering, National Sun Yat-Sen University, Kaohsiung, Taiwan; ^6^ Department of Healthcare Administration and Medical Informatics, Kaohsiung Medical University, Kaohsiung, Taiwan; ^7^ Institute for Translational Research in Biomedicine and Center for Shockwave Medicine and Tissue Engineering, Kaohsiung Chang Gung Memorial Hospital, Kaohsiung, Taiwan; ^8^ Department of Medical Research, China Medical University Hospital, China Medical University, Taichung, Taiwan; ^9^ Department of Nursing, Asia University, Taichung, Taiwan; ^10^ Department for Traditional Chinese Medicine and Center of Excellence for Chang Gung Research Datalink, Chang Gung Memorial Hospital, Chiayi, Taiwan; ^11^ Institute of Occupational Medicine and Industrial Hygiene, National Taiwan University College of Public Health, Taipei, Taiwan; ^12^ School of Traditional Chinese Medicine, College of Medicine, Chang Gung University, Taoyuan, Taiwan

**Keywords:** autosomal-dominant polycystic kidney disease, cerebrovascular accident, renin-angiotensin-aldosterone system blockade, statin, population-based cohort study

## Abstract

Fairly limited data reported the incidence and risk of cerebrovascular accident (CVA) in autosomal dominant polycystic kidney disease (ADPKD). Additionally, little is known regarding the therapeutic impact of renin-angiotensin-aldosterone system (RAAS) blockade and statin on reducing the occurrence of CVA in ADPKD. We utilized the data from Taiwan National Health Insurance Research Database (NHIRD) to perform a population-based cohort study (1997-2013). A total of 2,647 patients with ADPKD were selected from 1,000,000 general population after excluding patients with age<18, renal replacement therapy and concomitant diagnosis of CVA. Additionally, non-ADPKD subjects were assigned as comparison group by matching study cohort with age, gender, income and urbanization in 1:10 ratio (n=26,470). The results showed that ADPKD group had significantly higher frequency rate and cumulative incidence of CVA as compared with the non-ADPKD group (8.73% *v.s.* 3.93%, *p*<0.0001). Furthermore, the frequencies of both hemorrhagic and ischemic strokes were also significantly higher in the ADPKD than non-ADPKD group (all *p*-values <0.0001). After adjusting for age, gender and atherosclerotic risk factors with multivariate analysis, ADPKD independently carried 2.34- and 5.12-fold risk for occurrence of CVA and hemorrhagic stroke (95% CI: 2.02-2.72 and 4.01-6.54), respectively. Combination therapy [adjusted (a) HR=0.19, 95% CI: 0.11-0.31] was superior to either RAAS blockade (aHR=0.37, 95% CI, 0.28-0.5) or statin (aHR=0.44, 95% CI, 0.24-0.79) alone for reducing the CVA occurrence in the ADPKD population. In conclusion, ADPKD was associated with an increased risk of CVA occurrence. Combined RAAS blockade and statin therapy effectively reduces the risk of CVA in ADPKD.

## INTRODUCTION

Autosomal dominant polycystic kidney disease (ADPKD) is the most common hereditary kidney disease that affects 1/1,000 to 1/400 individuals worldwide [1, 2], and accounts for 8-10% of patients with end-stage renal disease (ESRD) in the Western countries [3]. In addition, these patients have been found around threefold mortality rate compared with general population [4] if lacks of appropriate treatment. Furthermore, not only multiple cystic expansions throughout the renal parenchyma but also extrarenal involvement with cystic and noncystic manifestations have been well identified [5]. Of importance is that the vascular complications have been demonstrated as the leading cause of death in patients with ADPKD [6].

The prevalence of intracranial aneurysm (ICA) in ADPKD has been established from 4% to as high as 41% with a positive family history [3, 7]. Additionally, a review from Cagnazzo et al. revealed that approximately 11% of ADPKD population have unruptured intracranial aneurysms and the rupture rate appears comparable to that of the general population [8]. Furthermore, 30-day mortality rate of subarachnoid hemorrhage (SAH) from rupture of ICA has been reported to be up to 45% and the survivors have about 30% chance of moderate to severe disability [9]. Therefore, current guidelines [10] have recommended to carefully screen ADPKD patients with family history of ICA or SAH, previous ICA rupture, and high risk professions. Recently, Yoo et al. has further reported that the patients with ADPKD on maintenance dialysis have around threefold risk for intracranial hemorrhage (ICH) [11]. However, there is still lack of information regarding real-world incidence and risk of cerebrovascular accident (CVA) in the ADPKD patients with preservation of complete or partial renal function. Furthermore, feasible therapeutic modalities that could reduce the occurrence of CVA in ADPKD remain unclear.

By using the 17-year Taiwan National Health Insurance Research Database (NHIRD) [12], we intended to study the real-world incidence and associated risk of CVA, including ischemic and hemorrhagic strokes, in Asian ADPKD patients. Besides, abundant data have demonstrated that Renin-angiotensin-aldosterone system (RAAS) blockade and statin (3-hydroxy-3-methylglutaryl-coenzyme A reductase inhibitor) are two standard therapies not only for primary and secondary preventions but also for improving the short-term and long-term outcome of cardiovascular accidents [13–17]. These two medications are recommended by current consensus guidelines [18] to treat ADPKD-accompanying hypertension and dyslipidemia. Accordingly, we also intended to assess whether administration of RAAS blockade or statin in the ADPKD population could decrease the risk of CVA occurrence.

## RESULTS

### Demographic characteristics, comorbidities, medications, and frequency of CVA in patients with and without ADPKD (Table 1)

A total of 2,647 patients with ADPKD and 26,470 matched patients without ADPKD were eligible during 17-year dataset period. In both groups of ADPKD and non-ADPKD, 51.5% of patients were female and majority (90.3%) of their age was between 18 and 65 with a median of 46 years old (interquartile range 37-55). Except for diabetes mellitus (17.76% *v.s.* 19.21%, *p*=0.069), the frequency rate of ADPKD-associated medical diseases (refer to Table 1) was significantly higher in the ADPKD than non-ADPKD group (all *p*-values <0.0001). Additionally, 60.26% and 26.52% of ADPKD patients were on the treatment with RAAS blockade and statin, respectively, which had significantly higher prescription rate than general population (both *p*-values <0.0001).

**Table 1:** Demographic data and frequency of CVA in patients with and without ADPKD

	ADPKD (N = 2647)		Non-ADPKD* (N = 26470)		P-value^a^
	No.	%	No.	%	
Gender					1.00
Female	1362	51.45	13620	51.45	
Male	1285	48.55	12850	48.55	
Age					1.00
18-39	808	30.53	8080	30.53	
40-65	1582	59.77	15820	59.77	
>65	257	9.71	2570	9.71	
Median age (IQR)	46 (37-55)		46 (37-55)		
Medical diseases					
Hypertension	2230	84.25	9764	36.89	<.0001
Diabetes mellitus	470	17.76	5085	19.21	0.069
Dyslipidemia	1102	41.63	7862	29.70	<.0001
Gout	834	31.51	3656	13.81	<.0001
Cardiac dysrhythmia	436	16.47	2890	10.92	<.0001
Atrial fibrillation	90	3.40	516	1.95	<.0001
Chronic ischemic heart disease	759	28.67	4618	17.45	<.0001
Heart failure	327	12.35	1306	4.93	<.0001
Peripheral vascular disease	209	7.90	1135	4.29	<.0001
Chronic kidney disease	1572	59.39	908	3.43	<.0001
Medications					
RAAS blockade					<.0001
No	1052	39.74	21474	81.13	
Yes	1595	60.26	4996	18.87	
Statin					<.0001
No	1945	73.48	22841	86.29	
Yes	702	26.52	3629	13.71	
CVA	231	8.73	1041	3.93	<.0001
Hemorrhagic stroke	119	4.48	245	0.92	<.0001
Subarachnoid hemorrhage	36	1.36	30	0.11	<.0001
Intracranial hemorrhage	95	3.59	216	0.82	<.0001
Ischemic stroke	134	5.04	860	3.23	<.0001

*Control group (non-ADPKD group) was matched by age, sex, taxable income and urbanization level.

^a^Chi-square test for categorical variables.

CVA = cerebrovascular accident, ADPKD = autosomal-dominant polycystic kidney disease,

IQR = interquartile range, RAAS = renin-angiotensin-aldosterone system.

The average follow-up period in ADPKD and non-ADPKD group was 9.26±4.48 and 10.01±4.41 years, respectively. At the end of follow-up period, a total of 231 and 1041 cases of CVA occurred in 2,647 ADPKD and 26,470 non-ADPKD patients, respectively. Accordingly, the ADPKD group had significantly higher frequency rate of CVA as compared with the non-ADPKD group (8.73% *v.s.* 3.93%, *p*<0.0001). Likewise, the frequencies of both hemorrhagic and ischemic strokes were also significantly higher in the ADPKD than non-ADPKD group (all *p*-values <0.0001). However, in the ADPKD patients, the frequency rate between hemorrhagic and ischemic strokes was similar (4.48% *v.s.* 5.04%, *p*=0.161). It was noteworthy that 30% (36/119) of hemorrhagic stroke was subarachnoid hemorrhage and the other 70% (95/119) was intracranial hemorrhage.

### Comparison of incidence of CVA between ADPKD and non-ADPKD groups (Table 2)

Table 2 shows that the incidence rate of CVA in patients with and without ADPKD was 941.9 and 391.6 per 100,000 person-years, respectively. Therefore, incidence rate ratio (IRR) of CVA in ADPKD to non-ADPKD was 2.4 (95% CI 2.09 to 2.77, *p*<0.0001). Of note, the IRR of hemorrhagic stroke was significantly higher in the ADPKD than non-ADPKD group with a 5.2-fold ratio (95% CI 4.21 to 6.52, *p*<0.0001). On the contrary, the IRR of ischemic stroke was only 1.7. The finding indicated that the brain events in ADPKD population had a higher tendency toward intracranial bleeding than atherosclerotic occlusive or thromboembolic diseases.

**Table 2:** Comparison of incidence of CVA, including hemorrhagic and ischemic strokes, between patients with and without ADPKD

Variables	ADPKD (N = 2647)		Non-ADPKD (N = 26470)		IRR (95% CI)	P-value
	Event	Incidence rate	Event	Incidence rate		
CVA	231	941.9 (828.0-1071.6)	1041	391.6 (368.5-416.1)	2.4 (2.09-2.77)	<.0001
Hemorrhagic stroke	119	471.8 (394.2-564.7)	245	90.0 (79.5-102.1)	5.2 (4.21-6.52)	<.0001
Ischemic stroke	134	536.6 (453.0-635.6)	860	320.9 (300.2-343.1)	1.7 (1.39-2.01)	<.0001

Incidence rate denotes events per 100,000 person-years, IRR = incidence rate ratio,

CVA = cerebrovascular accident, ADPKD = autosomal-dominant polycystic kidney disease,

CI = confidence interval.

### Occurrence of CVA in relation to length of time since diagnosis of ADPKD (Figure 1)

**Figure 1:**
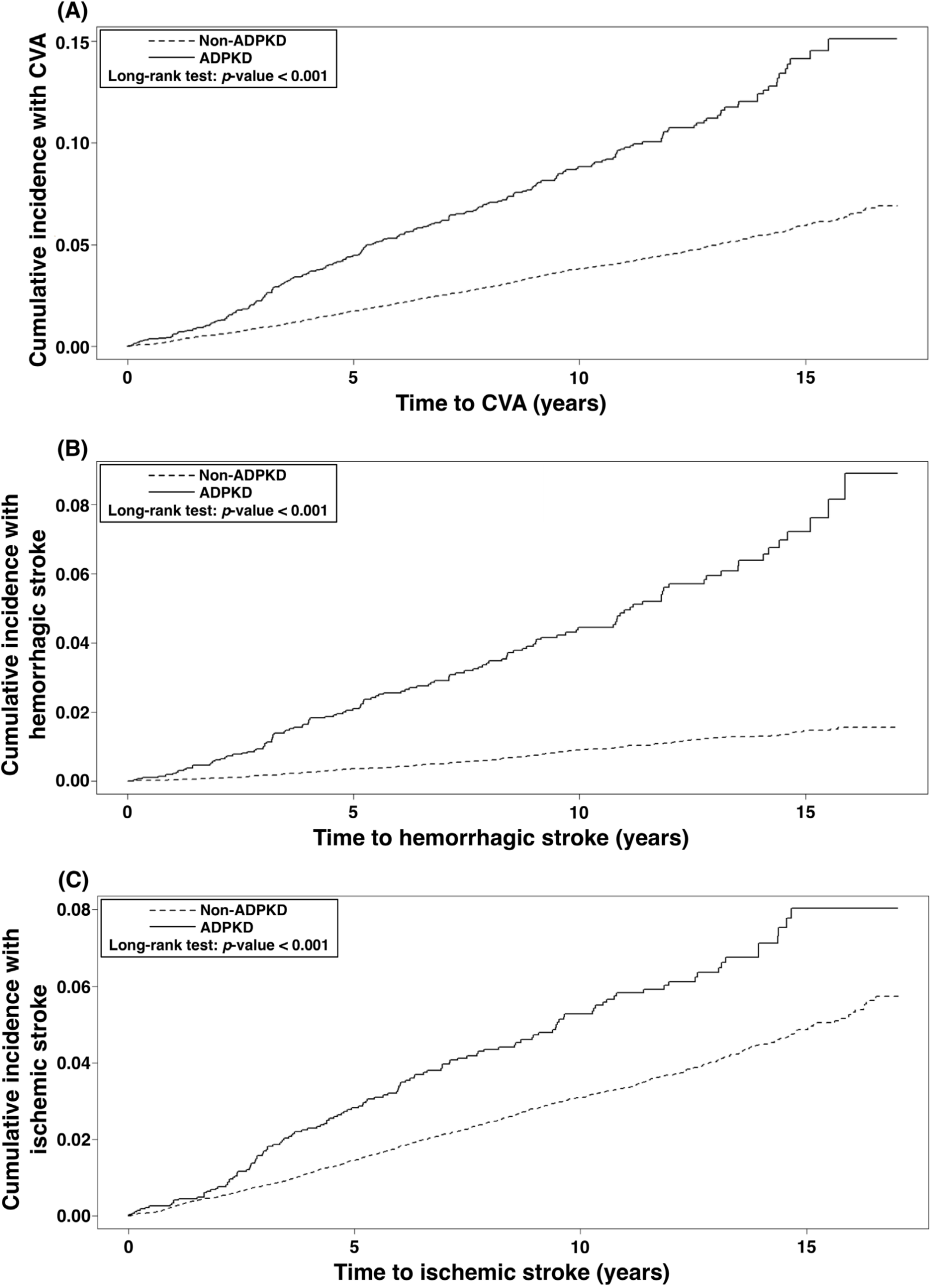
Cumulative incidence of CVA **(1A)**, hemorrhagic stroke **(1B)**, and ischemic stroke **(1C)** in the ADPKD versus the non-ADPKD group. ADPKD = autosomal dominant polycystic kidney disease, CVA = cerebrovascular accident.

Figure 1 demonstrates the Kaplan-Meier survival curves for cumulative incidence of CVA (1A), including hemorrhagic (1B) and ischemic (1C) strokes in patients with and without ADPKD. The results of Log-Rank test showed that ADPKD patients had significantly higher cumulative incidence of CVA than non-ADPKD counterparts (*p*<0.001). Similarly, cumulative incidences of hemorrhagic and ischemic strokes were also significantly higher in the ADPKD than non-ADPKD group (both *p*-values <0.001).

### Cox regression analysis for identification of the independent risk factors of CVA, including hemorrhagic stroke (Table 3)

By using the univariate Cox regression analysis, the risk of CVA significantly increased with age. Additionally, male gender, ADPKD, and each of ADPKD-associated medical diseases were also identified as the risk factors for CVA. After adjusting for age, gender, traditional atherosclerotic risk factors, i.e., hypertension, diabetes, and dyslipidemia, and medications with multivariate model, male gender, age older than 40, ADPKD, hypertension and diabetes remained powerful independent predictors for future development of CVA. Of these atherosclerotic risk factors, hypertension and elderly age (>65 years) were the two strongest independent risk factors for CVA occurrence. On the other hand, we found use of either RAAS blockade or statin protected against CVA. Moreover, combination therapy with RAAS blockade and statin was identified as a more powerful protective factor against CVA with an adjusted hazard ratio (aHR) of 0.22 (95% CI 0.17 to 0.27).

**Table 3:** Cox proportional hazard regression analysis for the risk of CVA and hemorrhagic strokes

	CVA				Hemorrhagic stroke			
	Univariate		Multivariate*		Univariate		Multivariate*	
	HR	95% CI	HR	95% CI	HR	95% CI	HR	95% CI
Gender								
Female	1.00		1.00		1.00		1.00	
Male	1.64	1.46 -1.83	1.65	1.47 - 1.86	1.72	1.39 - 2.12	1.69	1.36 - 2.10
Age								
18-39	1.00		1.00		1.00		1.00	
40-65	4.10	3.31 -5.08	3.36	2.69 - 4.20	2.31	1.70 - 3.14	2.17	1.57 - 3.01
>65	16.13	12.90 -20.16	7.71	6.09 - 9.76	5.14	3.61 - 7.32	3.18	2.17 - 4.65
ADPKD								
NO	1.00		1.00		1.00		1.00	
Yes	5.63	4.86 - 6.53	2.34	2.02 - 2.72	5.33	4.27 - 6.65	5.12	4.01 - 6.54
Medical diseases								
Hypertension	5.73	4.98 -6.59	4.95	4.21 - 5.80	4.50	3.51 - 5.78	4.54	3.39 - 6.09
Diabetes mellitus	1.99	1.77 -2.24	1.37	1.21 - 1.55	1.16	0.91 - 1.49	1.05	0.81 - 1.36
Dyslipidemia	1.34	1.20 -1.50	1.05	0.93 - 1.20	0.80	0.64 - 1.01	0.73	0.57 - 0.95
Gout	1.42	1.24 -1.63			1.26	0.97 - 1.63		
Cardiac dysrhythmia	2.00	1.75 -2.29			1.37	1.04 - 1.82		
Atrial fibrillation	3.73	3.05 -4.58			1.79	1.07 - 3.00		
Chronic ischemic heart disease	2.07	1.85 -2.33			1.28	1.00 - 1.63		
Acute myocardial infarction	2.29	1.88 -2.80			1.33	0.83 - 2.14		
Heart failure	2.43	2.07 -2.85			1.95	1.41 - 2.70		
Peripheral vascular disease	1.55	1.26 -1.90			1.04	0.66 - 1.65		
Chronic kidney disease	2.18	1.88 -2.52			3.40	2.67 - 4.32		
Medications								
No RAASb & statin	1.00		1.00		1.00		1.00	
Statin alone	0.92	0.72 - 1.17	0.39	0.30 - 0.50	0.60	0.35 - 1.03	0.34	0.19 - 0.60
RAASb alone	1.42	1.24 - 1.63	0.39	0.33 - 0.45	1.47	1.15 - 1.89	0.37	0.29 - 0.49
RAASb & statin	0.83	0.67 - 1.01	0.22	0.17 - 0.27	0.49	0.30 - 0.79	0.16	0.10 - 0.27

*Multivariate analysis was done with adjustment for age, gender, hypertension, diabetes, dyslipidemia and medications.

CVA = cerebrovascular accident, HR = hazard ratio, CI = confidence interval,

ADPKD = autosomal dominant polycystic kidney disease, RAASb = renin-angiotensin-aldosterone system blockade.

Because hemorrhagic stroke existed an IRR of 5.2 in ADPKD to non-ADPKD, we did further Cox regression analysis for hemorrhagic stroke. After multivariate analysis with adjustment of the aforementioned potential confounders, ADPKD and hypertension were identified as the two strongest independent risk factors for occurrence of hemorrhagic stroke with the aHR of 5.12 and 4.54 (95% CI 4.01 to 6.54 and 3.39 to 6.09), respectively. Unsurprisingly, combined RAAS blockade and statin also effectively attenuated the occurrence of hemorrhagic stroke (aHR 0.16, 95% CI 0.1 to 0.27).

### Cox regression analysis for identifying the independent risk factors of CVA in the ADPKD patients (Table 4)

We further evaluated individual determinant factors of CVA and protective effects of RAAS blockade and statin on CVA occurrence in ADPKD patients. After adjusting for age, gender and atherosclerotic risk factors and medications with multivariate analysis, we found that male gender, subjects with age older than 40, and hypertension were independently predictive of CVA in ADPKD. In contrast, RAAS blockade and statin were found to be independently predictive of freedom from CVA in ADPKD. Furthermore, combination therapy of these two drugs offered the strongest protection against CVA occurrence in the ADPKD population (aHR 0.19, 95% CI 0.11 to 0.31).

**Table 4:** Cox proportional hazard regression analysis for the risk of CVA in patients with ADPKD

	Univariate		Multivariate*	
	HR	95% CI	HR	95% CI
Gender				
Female	1.00		1.00	
Male	1.34	1.03 - 1.73	1.36	1.04 - 1.77
Age				
18-39	1.00		1.00	
40-65	2.12	1.49 - 3.01	1.89	1.31 - 2.73
>65	4.48	2.88 - 6.97	3.30	2.08 - 5.25
Medical diseases				
Hypertension	2.63	1.50 - 4.60	3.88	2.16 - 6.96
Diabetes mellitus	1.21	0.88 - 1.66	1.12	0.80 - 1.55
Dyslipidemia	0.64	0.49 - 0.85	0.77	0.57 - 1.05
Medications				
No RAASb & statin	1.00		1.00	
Statin alone	0.52	0.30-0.91	0.44	0.24-0.79
RAASb alone	0.49	0.37-0.65	0.37	0.28-0.50
RAASb & statin	0.23	0.15-0.37	0.19	0.11-0.31

*Multivariate analysis was done with adjustment for age, gender, hypertension, diabetes,

dyslipidemia and medications.

CVA = cerebrovascular accident, ADPKD = autosomal-dominant polycystic kidney disease,

HR = hazard ratio, CI = confidence interval,

RAASb = renin-angiotensin-aldosterone system blockade.

### Effects of RAAS blockade and statin on the occurrence of CVA in the ADPKD patients (Figure 2)

**Figure 2:**
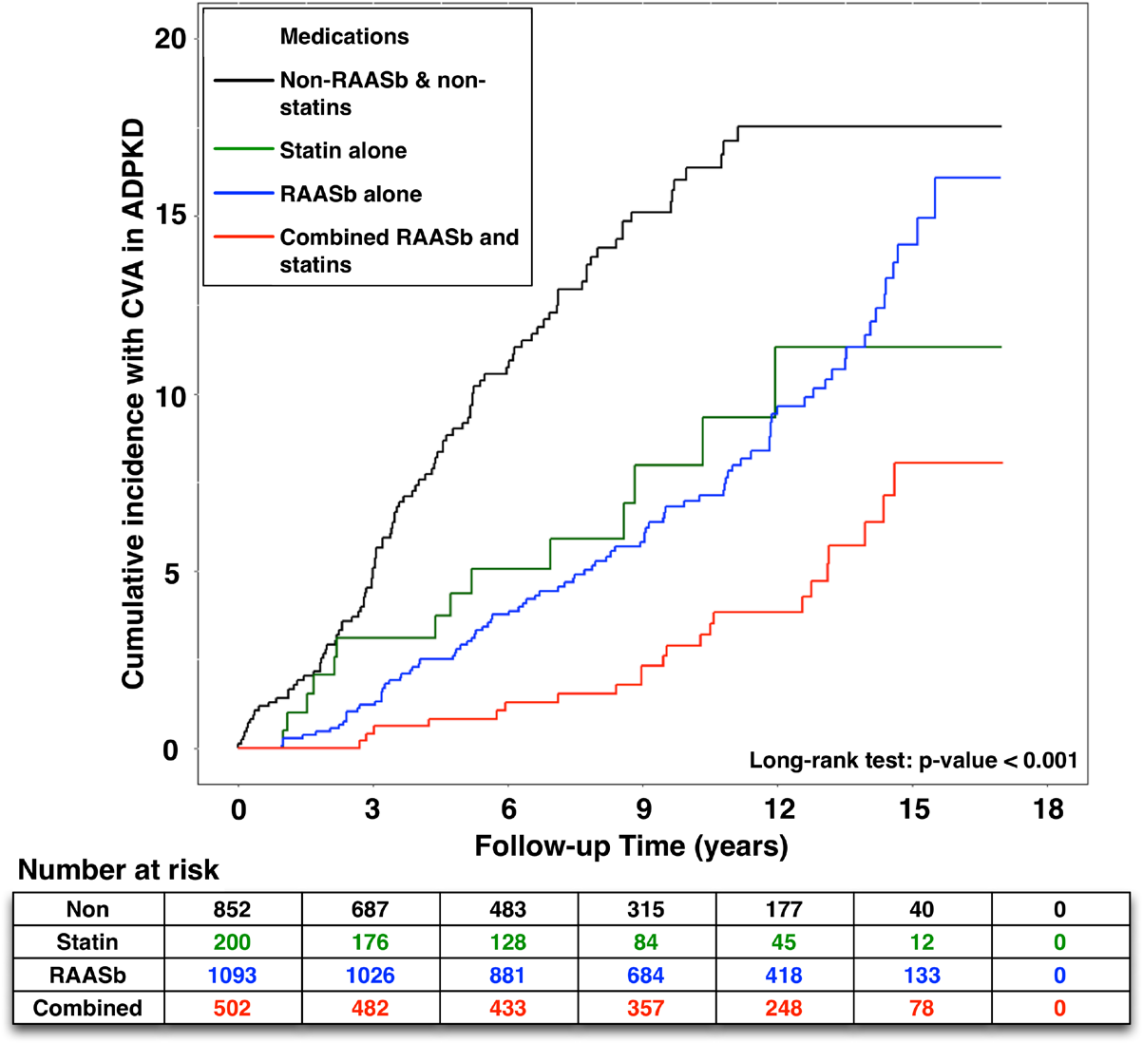
Effects of RAAS blockade and statin on the cumulative incidence of CVA in patients with ADPKD RAASb = renin-angiotensin-aldosterone system blockade, CVA = cerebrovascular accident, ADPKD = autosomal dominant polycystic kidney disease.

Figure 2 displays the Kaplan-Meier analysis for cumulative incidence of CVA in ADPKD with or without exposure to RAAS blockade and statin. We found that the cumulative incidence of CVA was reduced significantly with either one of the above treatment. Furthermore, the results of Log-Rank test revealed that ADPKD patients receiving combination therapy had the lowest cumulative incidence of CVA (*p*<0.001).

## DISCUSSION

The present study utilizing Taiwan NHIRD to investigate the association between ADPKD and CVA produced several striking clinical findings. First, the frequency and incidence of CVA, including hemorrhagic and ischemic strokes, were remarkably higher in the ADPKD than non-ADPKD population. Second, ADPKD independently carried an approximately 2- and 5-fold risk for occurrence of CVA and hemorrhagic stroke, respectively. Third, combination therapy of RAAS-blockade and statin, which were often prescribed for slowing down progression of chronic kidney disease [13–17] and cardiovascular diseases [19–23], effectively reduced the occurrence of CVA in ADPKD.

To the best of our knowledge, this was the first nationwide population-based cohort study to identify the potentially high risk of CVA, especially hemorrhagic stroke, in the ADPKD population and to document the therapeutic effects of either RAAS blockade or statin on prevention of CVA occurrence in ADPKD. In addition, the effectiveness of RAAS blockade on protection against development of CVA was similar to that of statin, implying that prescription of either RAAS blockade for hypertension or statin for dyslipidemia could offer an additional benefit of around 60% of relative risk reduction (RRR) on event rate of CVA in ADPKD. Of the most importance was that combination therapy of RAAS blockade and statin further decreased the risk of CVA occurrence with an approximately 80% of RRR.

An essential finding in the present study was that ADPKD patients had fivefold risk of hemorrhagic stroke compared with general population, suggesting potential complications of rupture of these ICAs or resulted from hypertension-caused intracranial hemorrhage in ADPKD [3, 7]. Intriguingly, recent study by Yoo et al. has shown that the patients with ADPKD on maintenance dialysis have around threefold risk for ICH [11]. Another finding in our study was that the frequency of CVA in Taiwanese ADPKD was 8.73%, which was very close to previous reported data (i.e., about 7.5%) from ADPKD registration in the United States [24]. Besides, we also noted that ADPKD carried around twofold risk for CVA occurrence. Therefore, the findings from Yoo *et al* [11]. and our present study raise the need to assess a safe and effective therapeutic option not only for the ADPKD *per se* but also for the associated risk factors of ICA and atherosclerotic vascular diseases to prevent occurrence of hemorrhagic and ischemic strokes.

Previous study has clearly identified that hypertension plays an important role on the occurrence of ischemic or hemorrhagic stroke in ADPKD [25]. In the present study, although higher ratio of use of RAAS blockade (60%) could be explained for the higher frequency of hypertension (84%) in the ADPKD group, the hazard ratio of RAAS blockade was only around 0.4 for occurrence of CVA after adjusting atherosclerotic risk factors, including hypertension. This finding implied the effect of RAAS blockade on the risk reduction of CVA in ADPKD might be beyond the benefit of lowering blood pressure [26]. Similarly, statin might also possess cardio-/cerebrovascular protection mainly through anti-inflammation, anti-apoptosis and improvement of endothelial cell function beyond lipid-lowering effect [27, 28]. Collectively, the underlying mechanism regarding how the RAAS blockade or statin worked on reducing the risk of CVA should be deserved further discussion and investigation.

Previous studies have shown that polycystin 1 and polycystin 2, two essential protein products of *PKD1* and *PKD2* genes, are expressed in vascular smooth muscle and endothelium [29, 30]. Mutations in *PKD1* or *PKD2* gene lead to pathological dolichoectasias (i.e., elongations and distentions of the arteries caused by weakening of the vessel walls) and dissections of vessels [29–31]. As a result, these vascular manifestations account for high frequency rate of cardiovascular abnormalities and ICAs in ADPKD [1, 32]. Besides, the degeneration of arterial medial layer plays a crucial role for arterial aneurysm and aneurysmal dissection/rupture [33, 34]. Also, the basic studies have revealed that inflammtion [35], reactive oxygen species (ROS)/oxidaitve stress [36], and RAAS [37] not only directly parcticiapte in the initiation of endotheial dysfunction and the propagation of arterial atherosclerosis, but also involve in process of arterial aneursym and weakening the arterial medial layer. Furthermore, an association between ADPKD and endothelial dysfunction/oxidative stress and cardiovascular/cerebrovascular events has been extensively investigated [15, 38]. The copious data also have shown RAAS blockade and statin therapy remarkably improve endothelial cell function [39] and suppress the oxidative stress [40, 41], leading to the prevention of cardiovascular/cerebrovascular events [19–23]. Taking these findings [19–23, 39–41] into consideration, the results of previous and our studies could shed light on the significant reduction of incidence of CVA in ADPKD by the RAAS blockade and statin therapy.

### Clinical implications

Since there is no clinically approved specific therapy for ADPKD [13], current consensus guidelines [10] recommend management of hypertension, renal function decline and renal complications to prevent the progression of ADPKD against ESRD. Despite a lot of proposed therapeutic modalities have been tentatively suggested based on different pharmaceutic mechanisms, e.g., tolvaptan (i.e., vasopressin receptor blocker), somatostatin (i.e., growth hormone inhibitor) or mTOR inhibitor (such as sirolimus) [42, 43] for reducing the progression and complications of ADPKD, however, the consensus and practical evidence remained lacking to support the safety and effectiveness of these therapeutic agents. Our findings based on the real-world data in Taiwan (i.e., NHIRD) identified that the therapeutic potential of RAAS blockade and statin for ADPKD patients may be feasible and practical to reduce the risk of CVA in ADPKD that is beyond their blood pressure and lipid lowering effects.

### Study limitations

Our study has limitations. First, detailed information in terms of personal history and lifestyle such as smoking, body mass index, and functional capacity are not provided by Taiwan NHIRD. Second, all the data in the current study have been registered with ICD-9-CM codes, and therefore further classification of disease status and determination of characteristics of disease lesion were impracticable. Third, the laboratory data are not available in NHIRD. Finally, we did not obtain medications other than RAAS blockade and statin from NHIRD. Thus, the synergistic effects or interactions among the potentially therapeutic modalities could not be completely clarified in the current study. Also, the protective effects on CVA from RAAS blockade or statin in the general population were not further analyzed and compared in the present study.

## MATERIALS AND METHODS

### Data source

The National Health Insurance (NHI) program provides health care to 99% of the 23.74 million population and links 97% of the hospitals and clinics in Taiwan (http://nhird.nhri.org.tw/en/) [12]. Researchers are able to register and claim data of 1,000,000 individuals systematically selected from all insured enrollees in the National Health Research Institute (NHRI) data bank. The NHI dataset included robust information regarding medical facilities, details of inpatient and outpatient orders, dental services, prescription of drugs, patient care provided by physicians, and the scrambled registration files, e.g., payment, regions, catastrophic illness, other than laboratory data. Diagnoses are entered in based on the International Classification of Diseases, 9^th^ Revision, Clinical Modification (ICD-9-CM). The Ethics Institutional Review Board of Chang Gung Memorial Hospital approved this study (No.201601127B1).

### Study population

**Figure 3:**
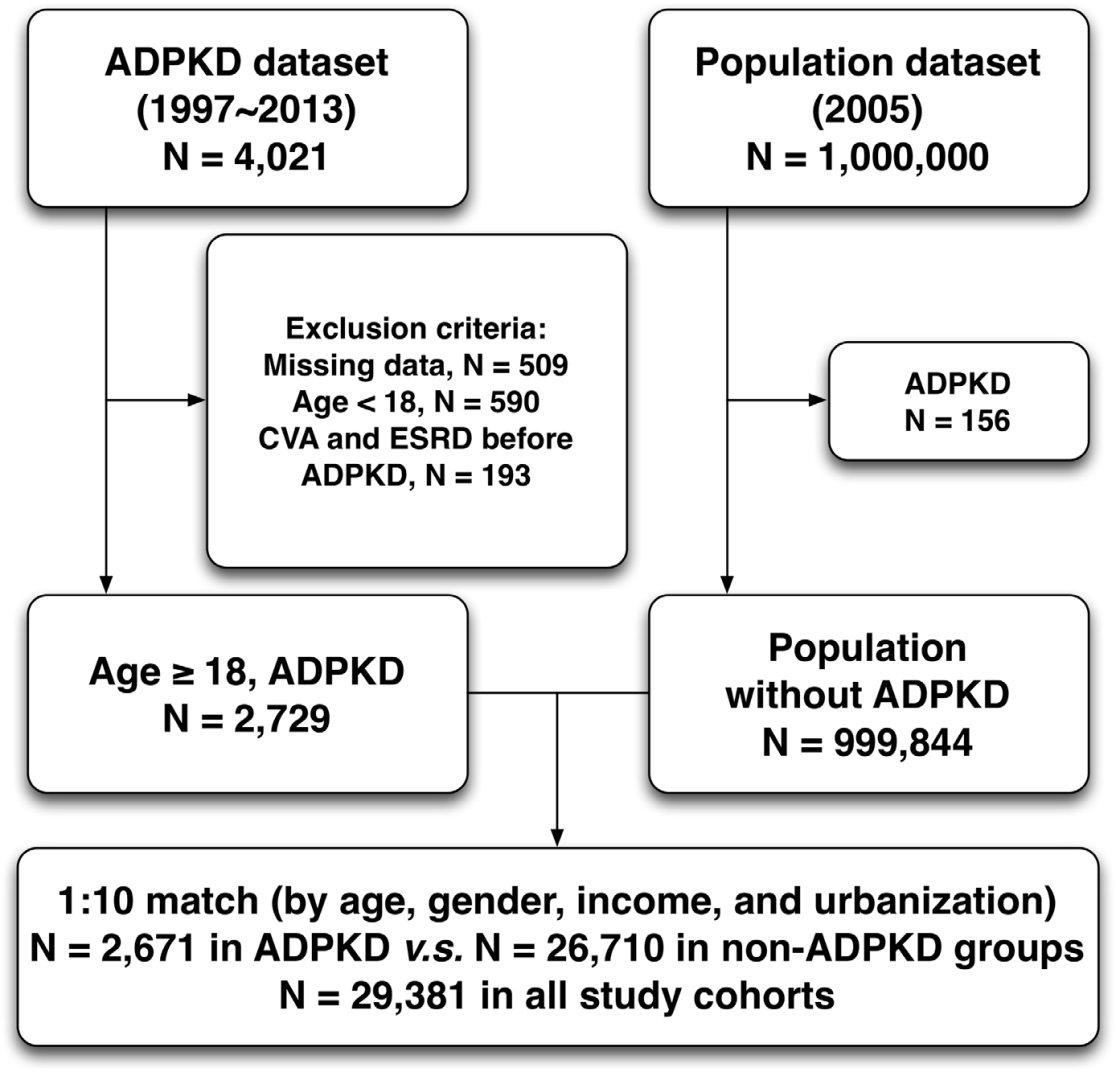
Flowchart of the patient enrollment for the ADPKD group and the matched non-ADPKD group ADPKD = autosomal dominant polycystic kidney disease, CVA = cerebrovascular accident, ESRD = end-stage renal disease.

This was a nationwide retrospective population-based cohort study. The diagnoses of ADPKD (ICD-9-CM codes 753.13) were confirmed by consecutive and at least three records of outpatient visits within one year or one diagnosis on admission during study period. We also verified the accuracy of diagnosis of ADPKD by checking the registration of catastrophic illness in NHIRD. The date of the initial diagnosis was defined as the index date. The follow-up period for each subject was evaluated from index date to the date of CVA (ICD-9-CM codes 430-436) occurrence, patient withdrawal from the insurance program, or the last date of the database. From January 1997 to December 2013, a total of 4,021 patients with ADPKD were identified from Taiwan NHRI databank. After excluding those patients with follow-up duration of less than one year, missing data on baseline characteristics, age of less than 18 years old, and initially concomitant diagnoses of CVA and end-stage renal disease (V451 and 549.8) at enrollment, a total of 2,647 ADPKD patients were identified as study cohort. The comparison cohort was selected randomly by age-, gender-, income-, and urbanization-matched individuals without history of ADPKD. The ratio of non-ADPKD to ADPKD was 10:1, and consequently 26,470 non-ADPKD patients were allocated into control group (Figure 3). The index date of the control group was the same as that of corresponding ADPKD patients. Besides, the duration of follow-up was similar in both ADPKD and non-ADPKD groups. Urbanization of the cities/counties was categorized into four levels, from level 1 to 4 indicating the most to the least urbanized degree, respectively. The insurance taxable income level per month (expressed by New Taiwan dollars, NTD) was also stratified into four classifications according to monthly salary of individual insured enrollee.

### Definition

We defined CVA as either an ischemic or a hemorrhagic stroke. Hemorrhagic stroke mainly included intracerebral hemorrhage and subarachnoid hemorrhage. Besides, RAAS blockade was composed of direct renin inhibitor, ACE inhibitor, ARB, or mineralocorticoid receptor antagonist. Exposure to RAAS blockade or statin was defined as the regimen duration longer than or equal to 3 months. On the contrary, non-exposure to RAAS blockade or statin was defined as those with no prescription of either one of them or regimen prescribed for less than 3 months. Short-term or temporary exposure to the above medications indicated prescriptions for transient discomfort on visiting, rather than standard treatment for ADPKD-associated medical diseases.

### Outcomes

We estimated pre-existing comorbidities for each participant with hypertension (ICD-9-CM codes 401-405), diabetes (250), dyslipidemia (272), gout (274), atrial fibrillation (427.31), ischemic heart disease (412-414, 429.2), acute myocardial infarction (410-411), heart failure (428), peripheral vascular disease (440, 443.9, 444.0, 444.2, 444.8, 444.9, 447.8, 447.9, 445.0, 445.02), chronic kidney disease (585), and malignancy of kidney or bladder (188-189).

The study outcome was a diagnosis of CVA (430-436) during the 17-year follow-up period. The purpose of this study was to evaluate the incidence and risk of CVA after the diagnosis of ADPKD. Besides, we investigated whether ADPKD was independently predictive of CVA. Furthermore, we analyzed the impact of RAAS blockade and statin on the risk of CVA and tested the hypothesis that the above treatment would effectively reduce CVA occurrence in ADPKD.

### Statistical analysis

We compared the distribution of demographic factors and the rate of comorbidities between the study cohort (i.e., ADPKD) and matched control cohort (i.e., non-ADPKD) with the independent *t* test or Chi-square test. The incidence rate and 95% confidence intervals (95% CI) of CVA were calculated for the entire follow-up period. We utilized the Kaplan-Meier method to estimate cumulative incidences and performed the Log-Rank test to examine differences between disease and non-disease groups or exposure and non-exposure groups in the cohort study. Furthermore, Cox proportional hazard regression models were used to compute the hazard ratios (HRs) and the accompanying 95% CIs after adjusting for age, gender, and associated risk factors. Interaction between RAAS blockade and statin was also performed for analyses of acting alone or combination effects on the risk of CVA occurrence. Two-tailed *p*-value <0.05 was considered statistically significant. All analyses were conducted using SAS statistical software (Version 9.4; SAS Institute, Cary, NC, USA).

## CONCLUSION

The results of this cohort study demonstrated that the frequency of CVA in ADPKD is similar between Asian and Western countries. ADPKD carries a two- and five-fold risk for occurrence of CVA and hemorrhagic stroke, respectively. Additionally, combination therapy of RAAS blockade and statin for ADPKD may have a therapeutic potential for the risk reduction of CVA.

## Abbreviations

RAAS: renin-angiotensin-aldosterone system
CVA: cerebrovascular accident
ADPKD: autosomal-dominant polycystic kidney disease
ESRD: end-stage renal disease
ICA: intracranial aneurysm
SAH: subarachnoid hemorrhage
ICH: intracranial hemorrhage
NHIRD: National Health Insurance Research Database
NHRI: National Health Research Institute
HRs: hazard ratios
